# Supplementary material for: Cancer cell-expressed SLAMF7 is not required for CD47-mediated phagocytosis
Source: Nat Commun. 2019 Feb 1;10:533. doi: 10.1038/s41467-018-08013-z (PMC6358615; doi:10.1038/s41467-018-08013-z)
Supplement: Supplementary file 1 — Description of Additional Supplementary Files [file 41467_2018_8013_MOESM1_ESM.docx]

**Title:** Supplementary Movie 1.
**Description:** Macrophages were mixed with V450-labelled cancer cells and incubated for 2 hours in the absence of stimulation. After treatment, non-adherent cancer cells were washed away and a Z-stack movie of CD11b-stained macrophages was performed.

**Title:** Supplementary Movie 2.
**Description:** Macrophages were mixed with V450-labelled cancer cells and incubated for 2 hours with CD47 antibody inhibrix at 5µg/ml. After treatment, non-adherent cancer cells were washed away and a Z-stack movie of CD11b-stained macrophages was performed.
