# Supplementary material for: Cancer cell-expressed SLAMF7 is not required for CD47-mediated phagocytosis
Source: Nat Commun. 2019 Feb 1;10:533. doi: 10.1038/s41467-018-08013-z (PMC6358615; doi:10.1038/s41467-018-08013-z)
Supplement: Supplementary file 2 — Supplementary Information [file 41467_2018_8013_MOESM2_ESM.pdf]

Supplementary Information File

Cancer cell expressed SLAMF7 is not required for CD47-mediated phagocytosis

He et al.

Supplementary Figure 1

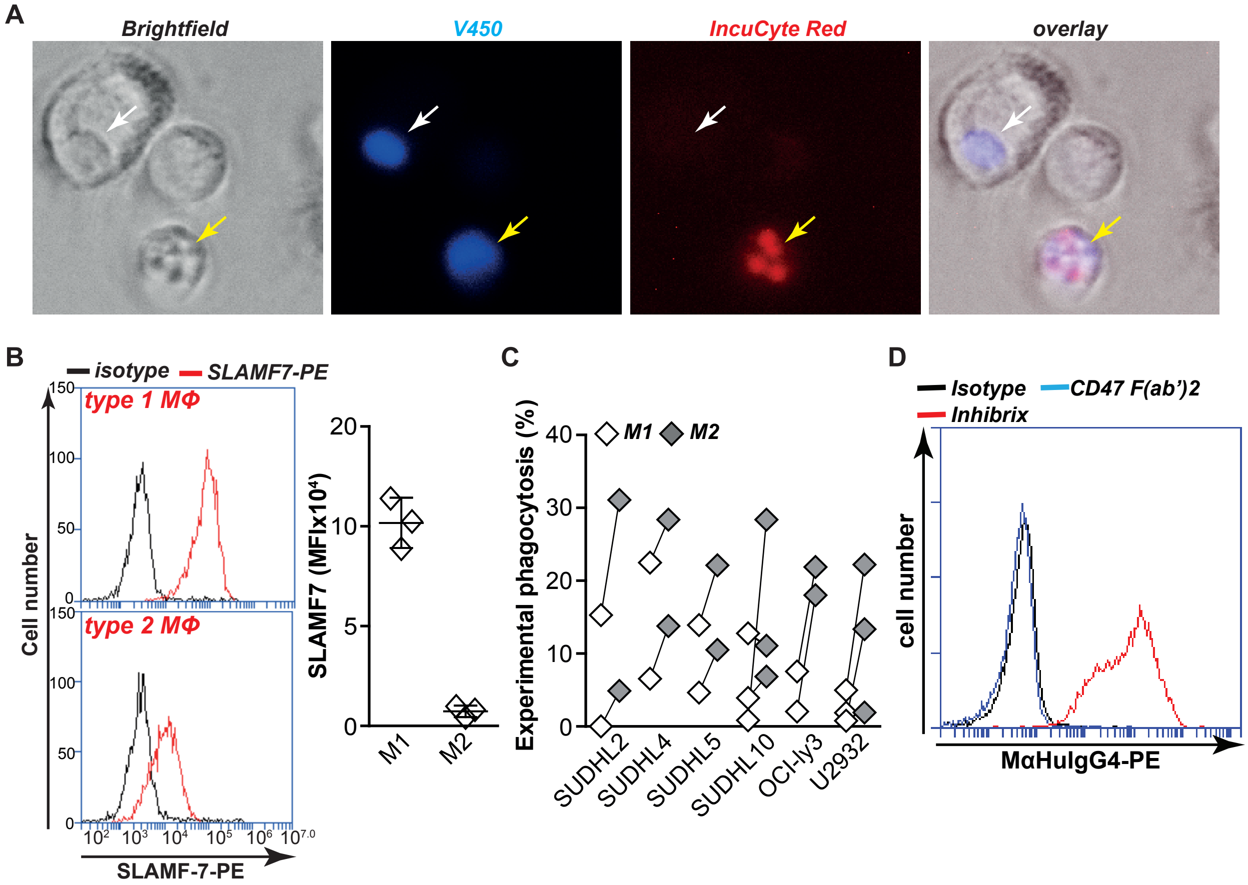

Supplementary Figure 1. A. V450 and Incucyte caspase-Red labelled cancer cells were incubated with macrophages for 2h, after which phagocytosis was assessed by fluorescent microscopy. ***White arrows:*** V450 and Incucyte caspase-Red negative cancer cells inside a macrophage. ***Yellow arrows:*** V450 and Incucyte caspase-Red positive apoptotic cancer cell that is not phagocytosed. **B.** SLAMF-7 expression level on both type 1 and type 2 macrophage derived from matched donors. **C.** Comparison of phagocytosis by CD47 F(ab')2 in 6 DLBCL lines between type 1 macrophage and type 2 macrophage (n=2). **D.** Validation of removal of the Fc domain from the CD47 F(ab')2 preparation. Presence of human IgG4 was assessed using PE-conjugated huIgG4 antibody after incubation of inhibrix en CD47 F(ab)'2 on CD47-expressing Jurkat cells, with no residual IgG4 staining after incubation with CD47 F(ab)'2.

Supplementary Fig.2

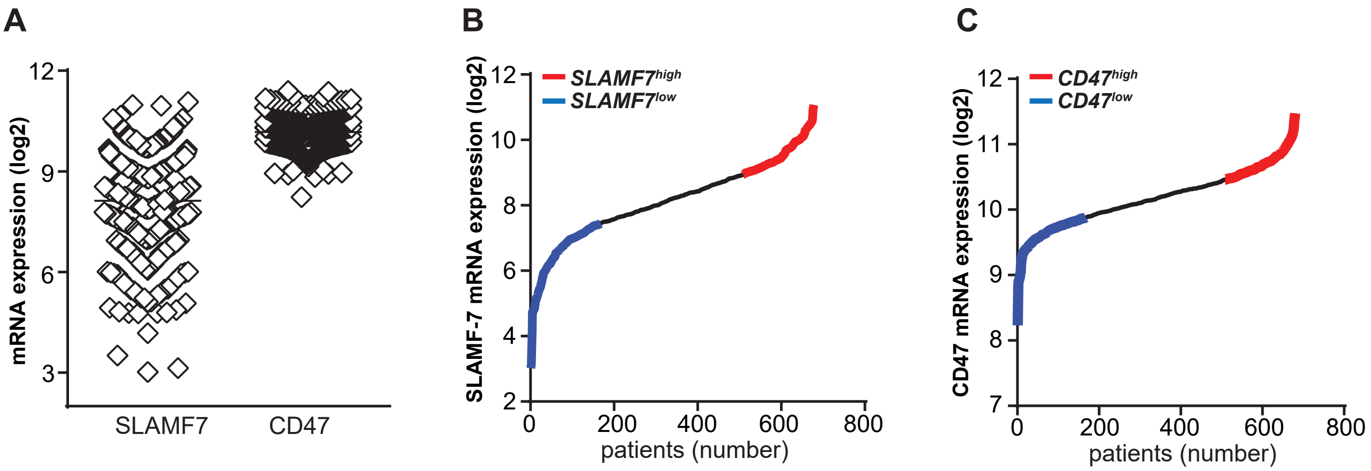

Supplemental Figure 2

A. SLAMF7 and CD47 gene expression profiles (log2) of 677 rituximab-cyclophosphomide-vincristin-doxorubicin-prednisone treated Diffuse Large B-cell lymphoma patients obtained from the public domain. **B.** Distribution of SLAMF7 expression, in blue SLAMF7 low expressing patients and in red SLAMF7 high expressing patients. **C.** Distribution of CD47 expression, in blue CD47 low expressing patients and in red CD47 high expressing patients.

Supplementary Fig. 3

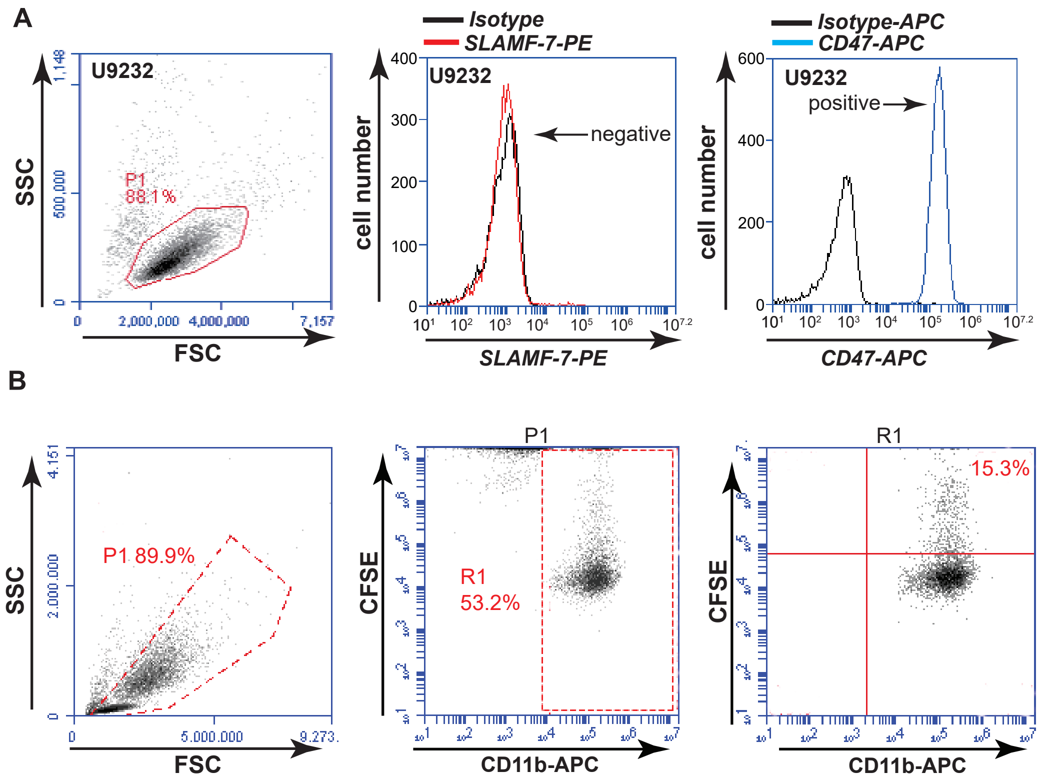

Supplementary Figure 3. A. Representative gating strategy for cell surface staining of SLAMF-7 (or CD47), as exemplifed using staining of U2932. **B.** Representative gating strategy for flow cytometric phagocytosis assay, using CFSE-labeled tumors and CD11b-labeled macrophages.

Supplementary Tables

Supplemental Table 1. Patient characteristics

**Variables**	**R-CHOP treated DLBCL patients (n=680)**
**Median age, years (range)**	63 (17-92)
**Gender**	
Male, n(%)	357 (58,2)
Female, n(%)	256 (37,6)
Unknown, n(%)	67 (9,9)
**COO**	
germinal center b cell (GCB), n(%)	305 (44,9)
Non-GCB, n(%)	375 (55,1)
**IPI categorized**	
Low risk (0,1)	235 (34,6)
Intermediate (2,3)	259 (38,1)
High risk (4,5)	71 (10,4)
Unknown, n(%)	115 (16,9)
**Ann Arbor**	
Non advanced stage of disease (1,2)	228 (33,5)
Advanced stage of disease (3,4)	255 (37,5)
Unknown, n(%)	197 (29,0)
**Median SLAMF7 expression, (range)**	8,20 (3,02-11,07)
**Median CD47 expression, (range)**	10,17 (8,23-11,47)

Supplemental Table 2. GEO accession numbers

**GSE_serie identifier**	**number of patients**	**Platform**	**PMID**					
GSE31312	409	GPL570	26324762	26248897	26111978	22437443	23982177	23775435
GSE4475	13	GPL96	16760442	23143595				
GSE10846	233	GPL570	19038878	21546504				
GSE23501	25	GPL570	20610814					
