# Supplementary material for: Cancer cell-expressed SLAMF7 is not required for CD47-mediated phagocytosis
Source: Nat Commun. 2019 Feb 1;10:533. doi: 10.1038/s41467-018-08013-z (PMC6358615; doi:10.1038/s41467-018-08013-z)
Supplement: Supplementary file 6 — Reporting Summary [file 41467_2018_8013_MOESM6_ESM.pdf]

nature research

Corresponding author(s): prof. dr. E. Bremer & dr. T. van Meerten

Reporting Summary

Nature Research wishes to improve the reproducibility of the work that we publish. This form provides structure for consistency and transparency in reporting. For further information on Nature Research policies, see Authors & Referees and the Editorial Policy Checklist.

Statistical parameters

When statistical analyses are reported, confirm that the following items are present in the relevant location (e.g. figure legend, table legend, main text, or Methods section).

n/a	Confirmed	
☐	☒	The exact sample size (*n*) for each experimental group/condition, given as a discrete number and unit of measurement
☐	☒	An indication of whether measurements were taken from distinct samples or whether the same sample was measured repeatedly
☐	☒	The statistical test(s) used AND whether they are one- or two-sided<br>*Only common tests should be described solely by name; describe more complex techniques in the Methods section.*
☒	☐	A description of all covariates tested
☐	☒	A description of any assumptions or corrections, such as tests of normality and adjustment for multiple comparisons
☐	☒	A full description of the statistics including central tendency (e.g. means) or other basic estimates (e.g. regression coefficient) AND variation (e.g. standard deviation) or associated estimates of uncertainty (e.g. confidence intervals)
☒	☐	For null hypothesis testing, the test statistic (e.g. *F*, *t*, *r*) with confidence intervals, effect sizes, degrees of freedom and *P* value noted<br>*Give P values as exact values whenever suitable.*
☒	☐	For Bayesian analysis, information on the choice of priors and Markov chain Monte Carlo settings
☒	☐	For hierarchical and complex designs, identification of the appropriate level for tests and full reporting of outcomes
☒	☐	Estimates of effect sizes (e.g. Cohen's *d*, Pearson's *r*), indicating how they were calculated
☐	☒	Clearly defined error bars<br>*State explicitly what error bars represent (e.g. SD, SE, CI)*

Our web collection on statistics for biologists may be useful.

Software and code

Policy information about availability of computer code

Data collection	Fluorescent microscopy pictures were analyzed using ImageJ. Flow cytometry data was acquired and analyzed using BD Accuri C6 CFlow software.
Data analysis	All analyses were conducted using SPSS statistics (version 23.0 Armonk, NY, IBM Corp.), STATA 14 (StataCorp LP, College Station, TX), and GraphPad Prism (GraphPad Prism [version 7.0b]; GraphPad Software, La Jolla, CA).

For manuscripts utilizing custom algorithms or software that are central to the research but not yet described in published literature, software must be made available to editors/reviewers upon request. We strongly encourage code deposition in a community repository (e.g. GitHub). See the Nature Research guidelines for submitting code & software for further information.

Data

Policy information about availability of data

All manuscripts must include a data availability statement. This statement should provide the following information, where applicable:

- Accession codes, unique identifiers, or web links for publicly available datasets
- A list of figures that have associated raw data
- A description of any restrictions on data availability

The datasets generated during and/or analysed during the current study are available from the corresponding author on reasonable request.

Field-specific reporting

Please select the best fit for your research. If you are not sure, read the appropriate sections before making your selection.

☒ Life sciences ☐ Behavioural & social sciences ☐ Ecological, evolutionary & environmental sciences

For a reference copy of the document with all sections, see nature.com/authors/policies/ReportingSummary-flat.pdf

Life sciences study design

All studies must disclose on these points even when the disclosure is negative.

Sample size	In respect of surface staining with anti-SLAMF-7 antibody, 0.5 million cells per condition were applied. Determination of cellular surface SLAMF-7 expression was repeated at least 3 times per cell line. The percentage phagocytosis was calculated by counting the number of macrophages containing V450-labeled tumor cells per 100 macrophages in 3 different fields-of-view per condition. The in vitro phagocytosis assay was performed with macrophages of at least 5 different healthy donors.
Data exclusions	No data were excluded from analysis
Replication	all attempts at replication were successful
Randomization	No randomization was performed. In each experiment, this study employed primary macrophages from one donor that were mixed with tumor cell lines. Thus, there were no factors requiring randomization.
Blinding	No blinding was necessary. Fluorescent microscopy and flow cytometry of the in vitro experiments were performed by experienced investigators.

Reporting for specific materials, systems and methods

Materials & experimental systems

n/a	Involved in the study
☒	☐ Unique biological materials
☐	☒ Antibodies
☐	☒ Eukaryotic cell lines
☒	☐ Palaeontology
☒	☐ Animals and other organisms
☒	☐ Human research participants

Methods

n/a	Involved in the study
☒	☐ ChIP-seq
☐	☒ Flow cytometry
☒	☐ MRI-based neuroimaging

Antibodies

Antibodies used	PE-labeled anti-human SLAMF7 antibody, Biolegend, cat.nr.331806, clone nr. 162.1, online data sheet and certificate for analysis: https://www.biolegend.com/en-us/products/pe-anti-human-cd319-cracc-antibody-4488<br>PE Mouse IgG2b, κ Isotype Ctrl Antibody, Biolegend, cat, nr. 400314, clone nr. MPC-11, online data sheet and certificate for analysis: https://www.biolegend.com/en-us/products/pe-mouse-igg2b--kappa-isotype-ctrl-1414<br>APC-labeled anti-CD3 antibody, Immunotools, cat nr.21810036, clone nr. HIT3b, online data sheet: http://www.immunotools.de/html/datas-apc/21810036.pdf<br>FITC-labeled anti-CD19 antibody, Immunotools, cat nr.21810193, clone nr HI19a, online data sheet: http://www.immunotools.de/html/datas-fitc/21810193.pdf<br><br>APC-labeled CD47 Monoclonal Antibody (B6H12), eBioscience™(Thermofisher), cat nr. 17-0479-42, clone nr. B6H12, online data sheet:<br>https://www.thermofisher.com/antibody/product/CD47-Antibody-clone-B6H12-Monoclonal/17-0479-42<br><br>FITC-labeled anti-CD20 antibody, eBioscience (Thermofisher), cat.nr. 11-0209-42, clone nr. 2H7, online data sheet: https://www.thermofisher.com/antibody/product/CD20-Antibody-clone-2H7-Monoclonal/11-0209-42<br>Anti-human CD47 IgG4 antibody (Inhibrix, clone B6H12 with human IgG4 domain) was generated by CRO Evitria
Validation	All antibodies are well characterized and validated by providers (see product sheets referred to above).

Eukaryotic cell lines

Policy information about cell lines

Cell line source(s)	OCI Ly3 DSMZ ACC761<br>U2932 DSMZ ACC633<br>SUDHL2 ATCC CRL2956<br>Karpas 422 DSMZ ACC32<br>SUDHL4 DSMZ AC495<br>SUDHL5 DSMZ ACC571<br>SUDHL6 DSMZ ACC572<br>SUDHL10 DSMZ ACC633<br>Raji ATCC CCL-86<br>Daudi ATCC CCl-21<br>RAMOS ATCC CRL-1596<br>BJAB ATCC ACC757<br>Z138 ATCC CRL 3001<br><br>DSMZ: Deutsche Sammlung from Microorganimen und Zellculturen, Braunschweig, Germany<br>ATCC: American Type Culture collection, Manassus, Virginia US
Authentication	STR profiling
Mycoplasma contamination	All cell lines were tested for mycoplasma contamination
Commonly misidentified lines<br>(See ICLAC register)	OCI-Ly3 was used as it is a DLBCL cell line. Identity was confirmed by STR profiling.

Flow Cytometry

Plots

Confirm that:

☒ The axis labels state the marker and fluorochrome used (e.g. CD4-FITC).

☒ The axis scales are clearly visible. Include numbers along axes only for bottom left plot of group (a 'group' is an analysis of identical markers).

☒ All plots are contour plots with outliers or pseudocolor plots.

☒ A numerical value for number of cells or percentage (with statistics) is provided.

Methodology

Sample preparation	All cell lines used in this study were cultured at 37°C in humidified 5% CO2 containing atmosphere.<br>Cells were harvested by centrifugation.<br>cells were resuspended in fresh medium with antibody.<br>Cells were incubated for 1h.<br>after 3 washes with cold PBS 3, cells were re-suspended in cold PBS<br>cells were measured with Accuri C6 flow cytometer
Instrument	BD Accuri C6 flow cytometer
Software	BD Accuri C6 CFlow software
Cell population abundance	no sorting was performed
Gating strategy	Viable cells were selected based on fsc/ssc, after which fluorescent intensity was analyzed for CD47 (using anti-CD47-APC) and SLAMF-7 (using SLAMF-7-PE). For strategy see Suppl. Fig. 3A.<br>For flow cytometric assessment of phagocytosis, mixed cultures were gated on macrophages in fsc/ssc, after which uptake V450-labelled cancer cells was quantified in FL1/FL4 dot-plot. For strategy see Suppl. Fig. 3B.

☒ Tick this box to confirm that a figure exemplifying the gating strategy is provided in the Supplementary Information.
